# An Unusual Presentation and Diagnosis of a Type 4 Middle Ear Paraganglioma: A Case Report

**DOI:** 10.7759/cureus.35358

**Published:** 2023-02-23

**Authors:** Noorullah Z Malik, Anthony Baumann, Deven Curtis, Avi Shaw, Anita Jeyakumar

**Affiliations:** 1 Biomedical Science, University of Akron, Akron, USA; 2 Department of Rehabilitation Services, University Hospitals Cleveland Medical Center, Cleveland, USA; 3 College of Medicine, Northeast Ohio Medical University, Rootstown, USA; 4 Otolaryngology - Head and Neck Surgery, Mercy Health, Youngstown, USA

**Keywords:** diagnosis, hearing loss, glomus tympanicum, otolaryngology, paraganglioma

## Abstract

We discuss the unusual presentation and subsequent diagnosis of a patient with a glomus tympanicum tumor, also known as middle ear paraganglioma, which is a rare and benign tumor of the middle ear that usually presents with pulsatile tinnitus, cranial nerve pathology, hearing loss, and dizziness. The patient was a 35-year-old female with no past medical or surgical history who presented with a three-year history of mild left-ear hearing loss. The initial examination was negative for otalgia or otorrhea but was notable for a mass filling her left ear canal. The patient denied pulsatile tinnitus or dizziness. CT imaging was used to examine the soft tissue mass in the middle ear and ear canal and was consistent with a soft tissue heterogeneous mass with a subsequent biopsy confirming a diagnosis of paraganglioma. After the diagnosis, a laser surgical excision was scheduled to remove the tumor. Constant awareness is needed to examine the different presentations of middle ear paragangliomas so that appropriate treatment is promptly provided.

## Introduction

Middle ear paragangliomas, also called glomus tympanicum tumors, are rare, benign, and slow-growing tumors that develop in the middle ear. These tumors are typically found in the area surrounding the cochlea and the vestibular system [1-3]. These paragangliomas are the most common primary tumor of the middle ear [4]. These tumors are thought to arise from the non-chromaffin sympathetic paraganglia, which are a group of cells found in various locations in the body, including the middle ear [1-3]. Glomus tympanicum tumors are classified into four types based on their size and location: type 1 (small mass limited to promontory), type 2 (tumor completely filling middle ear), type 3 (tumor filling middle ear and extending into the mastoid process), and type 4 (tumor filling middle ear, extending into mastoid or through tympanic membrane to fill external auditory canal; may extend anterior to the internal carotid artery) [1].

The symptoms of middle ear paraganglioma typically include hearing loss, dizziness, cranial nerve impairment, and pulsatile tinnitus [4]. Glomus tympanicum tumors most commonly occur in middle-aged women, but the overall incidence is incredibly rare [3,4]. High-resolution CT (HRCT) of the temporal bone is the imaging modality of choice for diagnosis [1]. After diagnosis, there are three treatment options for paragangliomas: radiotherapy, observation, and surgical removal; however, surgical removal is the main treatment option in many patients as increased growth of the paraganglioma can cause further hearing loss and symptomology [2-4]. Surgical removal involves many different options, including transcranial and endoscopic approaches with the use of a laser [3,5]. A hearing evaluation is often done before and after surgical treatment to help with diagnosis and monitor outcomes [2]. There is scarce data on type 4 middle ear paraganglioma tumors in the literature as most case reports focus on grade 1 or 2 paraganglioma tumors [2,5]. The overall incidence of each type of ear canal paraganglioma is unknown. The purpose of this case report is to highlight the unusual presentation and subsequent diagnosis of a type 4 glomus tympanicum/middle ear paraganglioma to enable better clinical recognition and appropriate patient care to improve overall outcomes.

## Case presentation

**Figure 1:**
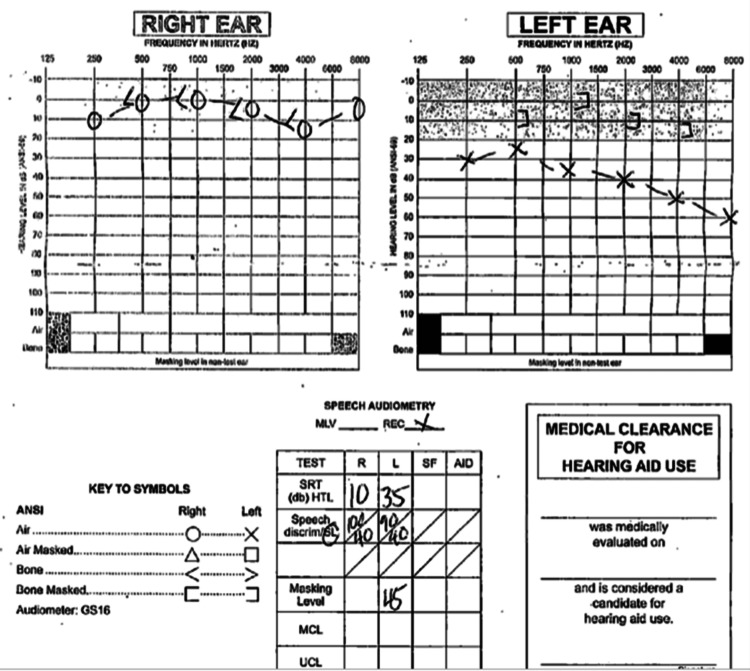
Pure tone audiogram performed at first office visit showing normal hearing of right ear with moderate conductive hearing loss in the left ear

A 35-year-old female presented to our clinic with a primary complaint of decreased hearing in the left ear over the past three years. She reported that the hearing in her left ear felt “muffled”. Due to her mild symptoms, she had initially presented to her family physician, who referred her to a general otolaryngologist. The general otolaryngologist had noted a mass filling the ear canal and referred her to an otologist. On evaluation, the patient was noted to have a smooth mass filling the ear canal and blocking any visibility of the tympanic membrane. An audiogram (Figure 1) showed left conductive hearing loss. Imaging revealed a heterogenous non-erosive soft tissue mass filling the middle ear space and extending into the ear canal. The differential diagnosis included polyp, granulation, cholesteatoma, mucocele, encephalocele, paraganglioma, and malignancy.

**Figure 2:**
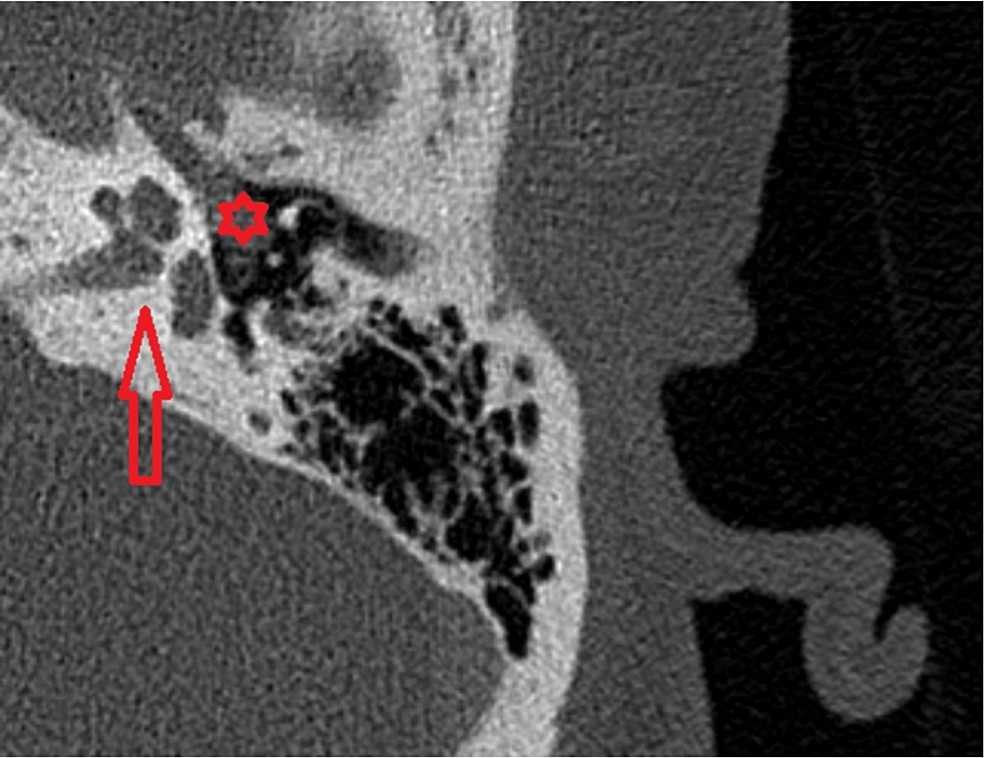
High-resolution axial CT IAC without contrast showing the left middle ear filled with soft tissue involving the incudostapedial joint CT: computed tomography; IAC: internal auditory canal

**Figure 3:**
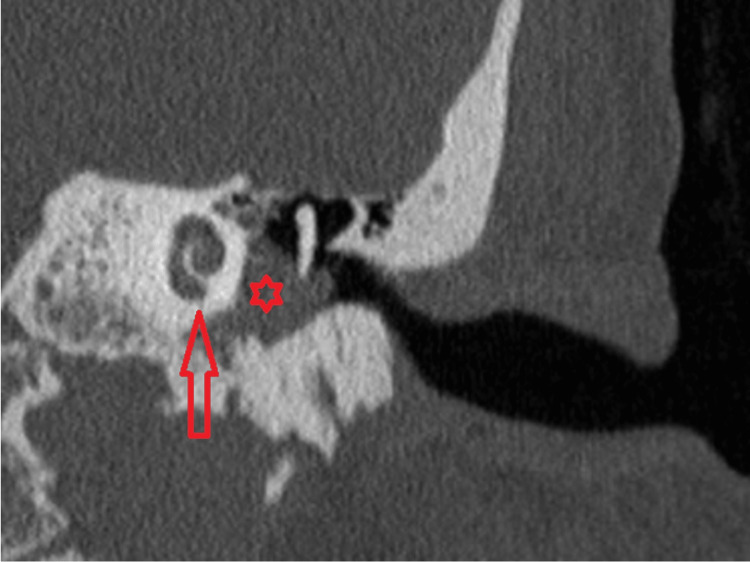
High-resolution coronal CT IAC without contrast showing the left middle ear filled with soft tissue CT: computed tomography; IAC: internal auditory canal

**Figure 4:**
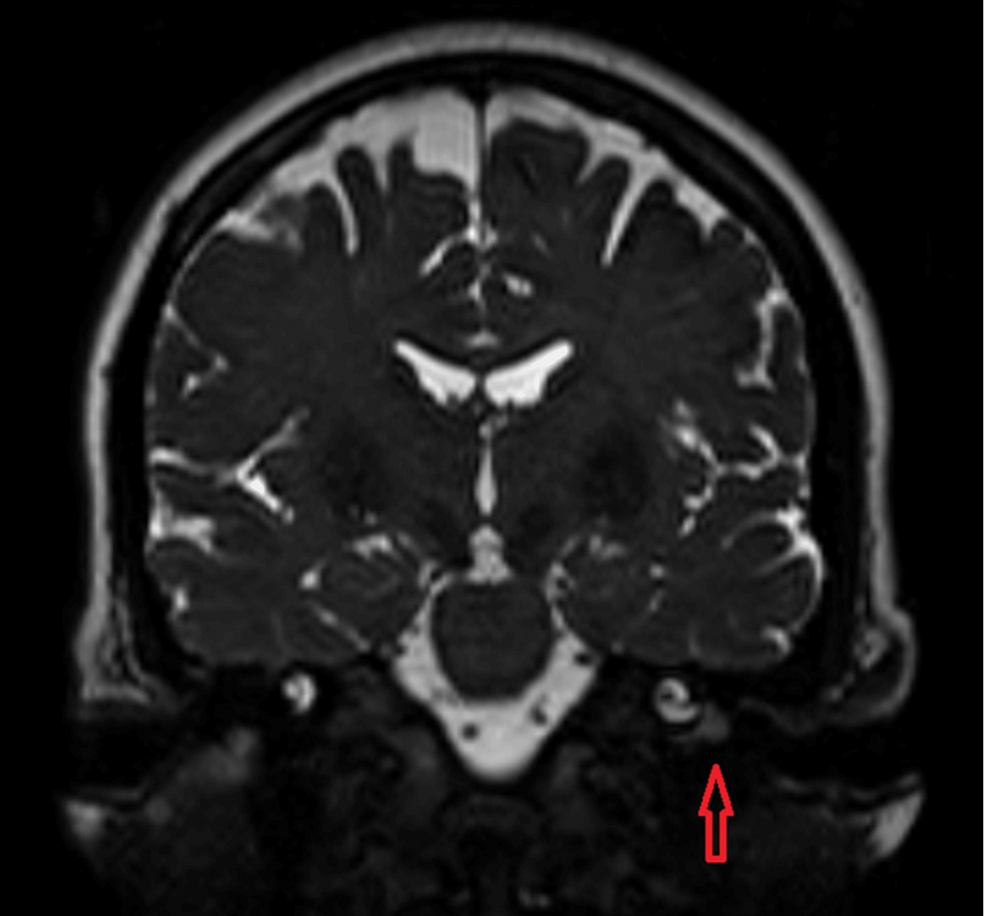
Coronal MRI T2 demonstrating an enhancing mass on the left cochlear promontory MRI: magnetic resonance imaging

Figures 2-4 illustrate the CT and MRI findings of the patient.

**Figure 5:**
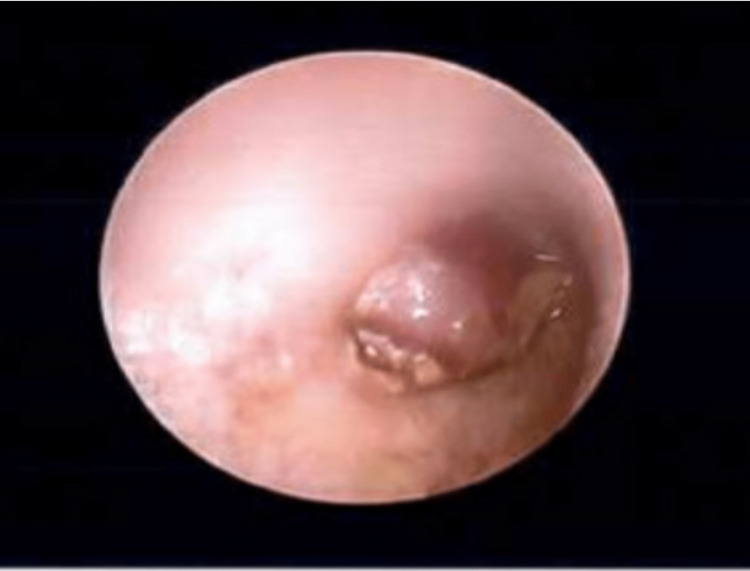
Otoscopic view of the left ear mass, no landmarks visible

**Figure 6:**
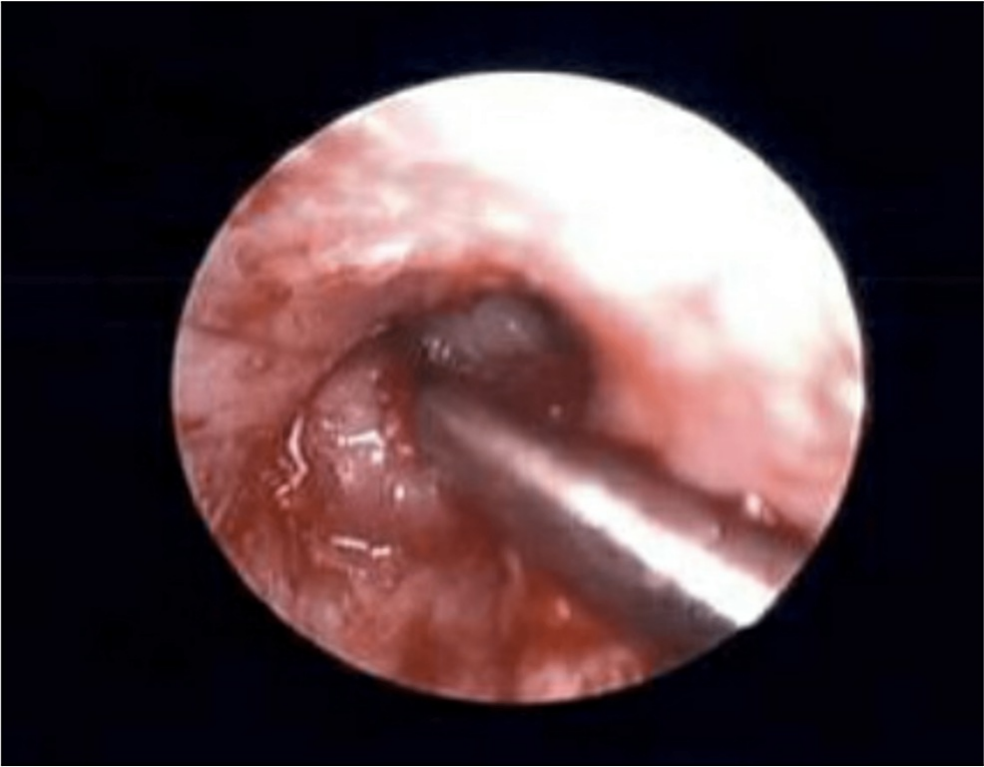
Otoscopic view of the left ear mass with a suction retracting the mass, with a small glimpse of the tympanic membrane distally

 The otoscopic view of the left ear mass is presented in Figure 5 and Figure 6.

**Figure 7:**
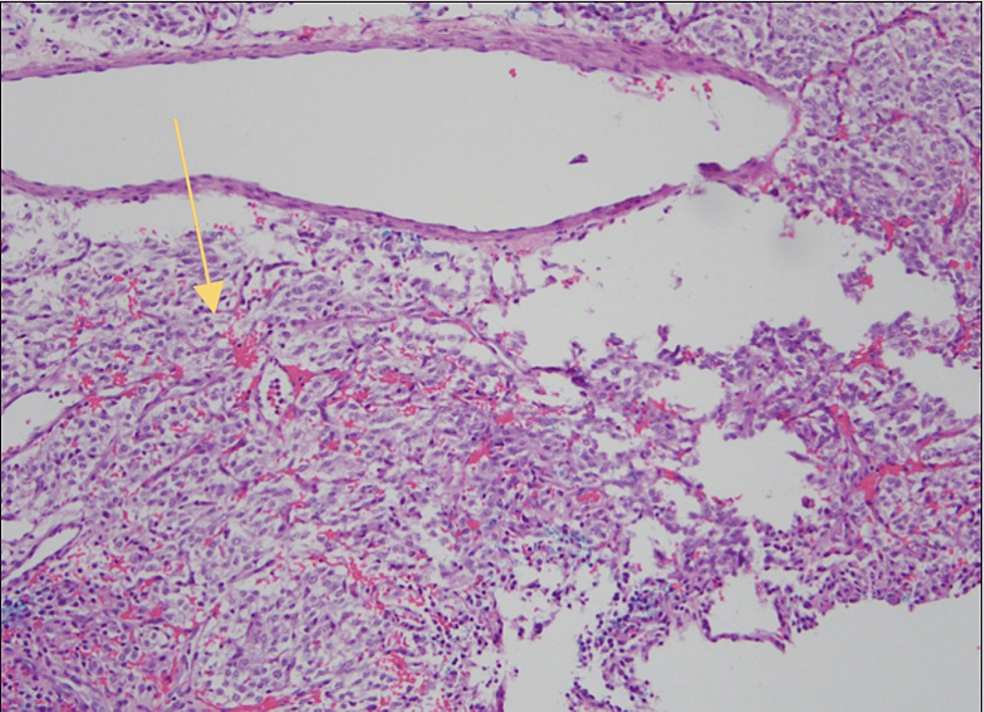
The histology of middle ear paraganglioma (light microscopy) Arrow pointing at histology consistent with middle ear paraganglioma

Due to the uncertain nature of the left middle ear mass, the patient was taken to surgery for a middle ear exploration and biopsy of the left middle ear mass. The biopsy was sent for pathological identification, and the pathology report subsequently revealed a left middle ear paraganglioma. Figure 7 depicts the histology of the middle ear paraganglioma.

Based on the size and location of the paraganglioma, it was classified as a type 4 glomus tympanicum [1]. A referral to endocrinology was made to examine other possible symptoms that typically present with paragangliomas, and the patient was scheduled for revision middle ear exploration with laser excision of the paraganglioma.

## Discussion

Middle ear paragangliomas, also called glomus tympanicum, are benign and highly vascular tumors that are rarely seen in clinical practice [4]. The incidence of the paraganglion tumor is said to be about one per 1.4 million people per year [3]. However, the relative rarity of middle ear paragangliomas can result in misdiagnosis and subsequent mistreatment, possibly leading to increased costs and adverse outcomes for affected patients. In patients with middle ear paragangliomas, the common symptoms include hearing loss, pulsatile tinnitus, and impairment of surrounding cranial nerves [4]. Our patient reported a three-year history of left ear hearing loss; however, further examination of the patient’s left ear revealed mild deficits in hearing loss consistent with conductive hearing loss. Small impairments in hearing could warrant evaluation and treatment by specialists. Furthermore, the patient did not have any tinnitus or facial nerve pathology, which are symptoms associated with middle ear paragangliomas [4].

In rare cases, paraganglioma tumors can increase the production of adrenaline, resulting in rapid heartbeat, headaches, and sweating [5]. In light of this, the patient was referred to endocrinology as interdisciplinary care could be helpful in managing the multiple distinct presentations of middle ear paragangliomas. As the presentation of this patient with middle ear paraganglioma was unusual, the physician believed that it was necessary to explore all avenues of symptomology to provide appropriate patient management. Although these lesions are histologically benign, they tend to be slow-growing and locally destructive [1,2]. Therefore, surgical excision is one of the most common treatment modalities, despite the paraganglioma’s benign nature. While paragangliomas rarely go on to progress to malignancy, surgical excision can stop local tissue destruction and help preserve hearing and vestibular function [4]. This case report highlights the treatment of a rare subtype of an uncommon benign middle ear tumor. Continued research is needed to determine the actual incidence of type 4 glomus tympanicum tumors, the long-term outcomes of surgical treatment, and the impact of different treatment strategies and surgical techniques on patient outcomes.

## Conclusions

Paragangliomas are benign, slow-growing tumors found throughout the body, including the middle ear. Although several different types exist, type 4 glomus tympanicum tumors are a rare type of paraganglioma found inside the middle ear. Most of the case reports in the literature focus on type 1 and type 2 middle ear paragangliomas. More research is needed to determine the incidence, examination, and treatment of type 4 paraganglioma tumors.
